# Adjuvant local antibiotic therapy in the management of diabetic foot osteomyelitis

**DOI:** 10.1186/s40842-024-00200-w

**Published:** 2024-12-16

**Authors:** Sara Metaoy, Iulia Rusu, Anand Pillai

**Affiliations:** 1 University of Manchester, Manchester, UK; 2 Wythenshawe Hospital Manchester, Manchester, UK

## Abstract

**Background:**

The management of diabetic foot osteomyelitis (DFO) is complex. The targeted use of adjuvant local antibiotics, in the form of biocomposite bone void filler, in DFO, can enhance patient outcomes while minimising the adverse effects associated with systemic antibiotic therapy and its shortcomings.

**Methods:**

We reviewed a series of 105 consecutive patients who underwent surgical management for diabetic foot osteomyelitis. In the NLAB group, (no adjuvant local antibiotic use), 49 patients, received the current standard of care treatment with no use of adjunctive local antibiotic therapy. In group LAB, (adjuvant use of local antibiotics), 56 patients received additional adjuvant local antibiotic therapy. Patient outcomes were compared between both groups.

**Results:**

Infection healing was demonstrated in 10 (20.41%) patients from group NLAB and 41 (73.21%) from group LAB (*p* < 0.0001). Persistence of infection with no evidence of wound healing, 6 months from surgery, was observed in 15 (30.61%) patients in group NLAB. Among the LAB group, only 4 (7.14%) patients demonstrated infection persistence (*p* = 0.00183). Reinfection was observed in 24 of 49 patients in group NLAB (49%) and in only 11 out of 56 patients in group LAB (20%) (*p* = 0.001466). 7 (6.67%) patients required major amputation with 6 (12.24%) belonging to group NLAB. Only 1 (1.78%) patient in group LAB underwent major amputation. A higher 5-year mortality rate was noted within patients in group NLAB, 27 (55.1%). The mortality rate in group LAB was (12.5%).

**Conclusion:**

The adjuvant use of antibiotic loaded bio-composite bone void filler locally was associated with increased infection clearance rates regarding diabetic foot osteomyelitis when compared with the standard care of treatment while achieving lower rates of infection persistence and recurrence. It also has the potential to reduce amputation and mortality rates with further research.

## Background

Diabetic foot ulcers (DFUs) are common, difficult to manage [1] diabetes-related complications, with a lifetime risk of ulcers developing between 19% and 34%.^2^ DFUs increase the risk of patients developing diabetic foot osteomyelitis (DFO), persistent infections, recurrent non-remitting infections, and increased overall mortality [2]. Management of DFO [3, 4] requires the involvement of members of a multidisciplinary team (MDT) (podiatrists, endocrinologists, infectious disease specialists, orthopaedic and vascular surgeons) [5] and ranges from antibiotic therapy, off-loading, and surgical interventions [6]. Most guidelines currently do not propose the use of local antibiotic therapy [3, 5, 7].

Antibiotic therapy is necessary for infection eradication with treatment duration ranging from 2 weeks to 6, varying according to severity of infection [6]. Empirical systemic antibiotics form the initial basis of management with more focused antibiotic therapy administered after assessment of culture results with guidance from microbiology [8]. Delayed wound healing, repeated infections by multi-drug resistant pathogens, and disease persistence are some of the factors affecting the overall efficacy of conventional systemic antibiotic therapy [9]. With recurrence rates as high as 44.5% 12 months post-surgical management of DFO, as reported by Schmidt [10], the focus of recent literature [11, 12] and studies has shifted towards the identification of novel therapeutic regimens to optimize complete DFO eradication [11] thereby reducing amputation rates and patient mortality, and enhancing overall patient outcome.

Some of the challenges associated with systemic antibiotic use in diabetic foot osteomyelitis include poor bone penetration, systemic toxicity with high antibiotic concentration administration [13], with repeated and prolonged antibiotic use increasing the length of hospital stay, predisposing patients to higher rates of reinfection [14] and adverse drug events [14]. Prolonged hospital stay, and increased patient morbidity have an impact on the overall financial burden and costs to the hospital. Pathogens causing DFO often display increased resistance to treatment through the formation of protective biofilms [11] in which systemic antibiotics are incapable of breaking down. This can lead to antibiotic resistance and tolerance.

Local antibiotic use in the management of diabetic foot osteomyelitis can help overcome the challenges associated with systemic antibiotic use, leading to superior patient outcomes [11, 12]. The delivery of antibiotics locally at the site of infection following surgical debridement allows higher concentrations to be administered with reduced potential for adverse effects [14, 15]. It also allows high levels of the antibiotics to be administered even in areas with compromised vasculature [16] overcoming obstacles caused by the presence of peripheral arterial disease (PAD) in patients with diabetic foot osteomyelitis. PAD can result in reduced blood flow and tissue ischaemia which decreases the bioavailability of systemic antibiotics [17].

There are numerous ways to deliver local antibiotics at the infection site and help prevent biofilm formation [18]. The use of antibiotic-loaded injectable bio-composite material [19] (Cerament®, Bonesupport, Lund, Sweden) was first developed for bone void filling and fracture stabilisation [20] and is now the topic of discussion for use in diabetic foot infections (DFIs) and DFO [21]. CERAMENT® is a synthetic bone void filler consisting of 40% hydroxyapatite, and 60% calcium sulfate [22] that provides structural support and promotes bone growth [22]. The addition of gentamicin or vancomycin to the void filler allows for the local delivery of high concentrations of the aforementioned antibiotics directly to the infected site [22, 23]. Antibiotics can be delivered locally at the wound site using several other modalities and carriers ranging from the use of chitosan gel [24], polymethylmethacrylate (PMMA) in the form of bone cement [25], to the use of antimicrobial peptides such as pexiganan and nisin [26].

The adjunctive use of local antibiotic delivery methods in DFO can in theory enhance infection eradication, reduce disease persistence and recurrence, and promote complete wound resolution.

## Methods and study sample

This is a retrospective single centre study undertaken at a teaching hospital in the northwest of England. Patients admitted to Wythenshawe hospital- Manchester University hospital for surgical management of diabetic foot osteomyelitis from 2019 to 2024 were identified (*n* = 105). All patients included in the study had Texas classification of B2/B3 and Sindbad scores of 4/5. DFO was diagnosed clinically and on the basis of deep samples/biopsy results, microbiology lab cultures, and review of x-ray and MRI scans. Soft tissue infections with no apparent bone involvement were excluded. Patients’ vascular status was assessed through review of ABPI measurements and arterial doppler studies. None of the patients required any vascular surgical intervention, and no patients in the study group had critical ischaemia.

All patients underwent podiatry treatment and dressings in the community, and oral antibiotics were administered by GPs prior to hospital attendance. All surgical procedures were carried out under the direction of one of our two fellowship trained foot and ankle surgeons with an interest in diabetic foot disease and an MDT involvement. One surgeon routinely uses adjuvant local antibiotic therapy in his practise. No other surgical procedures were performed exclusively.

We separated our cohort into two groups. The first group, NLAB, consisting of 49 patients, were offered the current standard of care treatment regimen for diabetic foot osteomyelitis which included IV antibiotics, off-loading using casts, and surgical interventions (ranging from debridement, minor amputation and major above/below the knee amputation).

Group LAB, consisting of 56 patients, was classified as patients who had undergone the same standard treatments as the patients in the NLAB group with the addition of adjuvant local antibiotic delivery through use of injectable bio-composite material, containing either gentamicin or vancomycin (CERAMENT®G /V), directly into the bone at the infection site following debridement with the majority receiving adjuvant Cerament G. The local antibiotic carrier bio-composite was applied intramedullary in forefoot applications and the silo technique employed in hindfoot applications [27].

Baseline patient characteristics including age, sex, HbA1c levels, WBC count, site of infection and surgical interventions, were collected and analysed through the use of the Electronic patients’ records (EPR) system. Patient outcomes were then classified in accordance with the IWGDF/IDSA guidelines as those experiencing healing with no residual signs of infection (clinically and on review of microbiology results), those experiencing infection persistence with no signs of healing and those experiencing recurrence of infection after a period of initial healing. We defined infection persistence as the presence of clinical signs of infection (induration, erythema, swelling) at the same wound site with no periods of resolution, and consistent evidence of growth of the same causative pathogen in subsequent/serial culture results for a period of 6 months from date of index surgery. Infection recurrence/ re-infection was defined as presence of a new infection at a different wound site or culture results demonstrating a different pathogen growing at the same site of infection, and any recurring infections following a period of healing of 3 months.

The outcomes for both groups were then recorded and compared and statistical significance (significant at *p* < 0.05) was tested for using the chi squared test and Fisher’s exact test for non-parametric categorical variables and the Mann-Whitney U test for continuous variables using the IBM SPSS software. Patients’ outcomes were independently reviewed and analysed and compared between both groups.

## Results

Our study involved a total of 105 patients, 49 classified as those belonging to the NLAB group and 56 classified as those belonging to the LAB group. In group LAB, 48 (85.71%) patients received CERAMENT G treatment while 8 (14.28%) received CERAMENT V (*n* = 56). CERAMENT V was indicated in predominantly gram-positive infections and in those with known gentamicin resistance. CERAMENT G was preferred when the pathogen was unknown as it allows for a more broad-spectrum cover. In group NLAB, 39 (79.59%) were male and 10 (20.4%) were female with an overall mean age of 63.21. In group LAB, 43 (76.78%) were male and 13 (23.21%) were female with a total mean age of 64.63. Demographic variables, age, sex, HbA1c levels, WBC count, and site of infection were matched between the two groups. This is demonstrated in Table 1. All patients in our cohort had undergone vascular studies (ABPI measurements and arterial doppler ultrasounds) to assess for any signs of peripheral arterial disease (PAD). Both groups displayed similar PAD rates with no vascular interventions needed within both groups. PAD was identified in group NLAB in 21 (42.85%) patients and in group LAB in 23 (41.07%) patients.

Culture samples from deep wound sites and bone were taken intra-operatively and the results were assessed for both groups. The gram positive bacteria, *Staphylococcus aureus*, was the most commonly identified pathogen in both sub-groups, 22 (44.89%) in group NLAB, 25 (44.64) in group LAB. Antimicrobial resistance patterns were studied for both groups. In group NLAB, 22 (44.89%) patients demonstrated antimicrobial resistance with Staphylococcus being resistant to clarithromycin in 8 (16.32%) patients. A similar pattern was noticed in patients in group LAB with a total of 24 (42.85) resistance patterns identified with Staphylococcus being resistant to clarithromycin in 10 (17.85%) patients. Out of the identified resistance patterns, the overall resistance to gentamicin was observed in 4 (3.81%) patients with 2 patients in group NLAB demonstrating resistance to gentamicin with recurrence of infection being observed in both patients. Similarly, gentamicin resistance was observed in 2 patients in group LAB both receiving CERAMENT G. One of those patients experienced infection recurrence while the other experienced complete wound resolution (50% infection recurrence in gentamicin resistance). There was no vancomycin resistance identified in our series.

Location of infection was classified descriptively as either the forefoot, mid-foot, or hind-foot. The forefoot was the most commonly affected site with 39 (79.59%) patients in group NLAB identified with presence of ulcers in their forefoot. Similarly, 40 (71.42%) patients in group LAB presented with infected ulcers in their forefoot. The mid-foot was affected in 4 (8.16%) patients in group NLAB and in 6 (10.71%) patients in group LAB. Hind-foot ulcers were represented in 6 (12.24%) patients in group NLAB and in 10 (17.85%) patients in group LAB.

The presence of osteomyelitis was confirmed clinically at time of surgery in all cases. Growth was obtained from bone samples at time of surgery in 40 (81.63%) patients in group NLAB and in 49 (87.5%) patients in group LAB. This may be due to several of the patients receiving pre-operative courses of antibiotics by their General Practitioners affecting sampling. The mean follow-up time for the entire cohort was 14 months (range 7–16) with both groups receiving existing standard of care and undergoing similar surgical procedures.

### Outcomes

Patients’ outcomes were recorded and classified into ulcer healing and infection eradication, infection persistence and infection recurrence. Complete infection eradication with no concurrent evidence of reinfection or disease persistence was demonstrated in 10 (20.41%) patients from group NLAB and 41 (73.21%) from group LAB (*p* < 0.00001, Chi-Square test). The use of adjuvant local antibiotic therapy was associated with improved infection eradication and reduced healing times. Average time for healing in group 1 was 6 months (range from 3 to 14), average time for healing for group 2 was 3 months (range from 2 to 6) with no statistical significance noted (*p* > 0.05).

In group NLAB, 15 (30.61%) patients demonstrated evidence of persistence of infection while only 4 (7.14%) patients in group LAB did (*p* = 0.00183, Chi-Square test).Reinfection was seen in 24 (48.97%) patients in group NLAB with an average time of recurrence of 6 months (range from 3 to14) and in group LAB reinfection was seen in 11 (19.64%) patients with an average time of recurrence of 8 months (range from 5 to 16). A statistically significant relationship was noted between the adjuvant use of local antibiotics and reduced re-infection rates (*p* = 0.001466, Chi-Square test). Patients’ outcomes are represented by Table 2.

We also accessed patients’ surgical notes and recorded the average number of procedures all patients in both sub-groups had undergone. Surgical procedures recorded ranged from wound debridement in theatre, minor amputations limited to the toes, and major amputations (above or below the knee) to Charcot foot reconstruction.

The average number of procedures patients in group NLAB had undergone was 2.44 (range from 1 to 9). The average number of procedures patients in group LAB had undergone was 2.25 (range from 1 to 4). Although our data shows the use of adjuvant local antibiotic therapy reduced the mean number of surgical procedures in comparison to no local antibiotic use, it did not reach statistical significance with a p score of 0.84148 using the Mann-Whitney U test.

A total of 7 patients demonstrated major treatment failure requiring amputation with 6 (12.24%) belonging to group NLAB. Only 1 (1.78%) patient in group LAB had undergone below the knee amputation. A statistically significant relationship between local antibiotic use and reduced amputation rates was noted (*p* = 0.0484, Fisher’s exact test).

Review of the overall mortality rates within both groups noted a statistically significant relationship between adjuvant use of local antibiotics and an overall reduction in mortality (*p* < 0.00001, Chi-Square test). A higher mortality rate was noted within patients in group NLAB, 27 (55.1%), with an average age of 64 at mortality. The mortality rate in group LAB was 7 (12.5%) with an average age of 63.71.

## Discussion

Diabetic foot infections pose significant challenges globally due to their complicated underlying pathophysiology, which is complicated by PAD, peripheral neuropathy, multidrug resistant pathogens, and biofilm formations. These factors predispose patients to disease persistence, repeated infections, and inadequate wound healing. Our study looked at the efficacy of local antibiotics in the scope of diabetic foot infections. We identified that there was a clear difference in patient outcome between groups with enhanced results observed in group LAB. The use of Cerament demonstrated more than a three-fold increase in healing rates and a reduced time to healing from 5 months to 3 months when compared with our patients in group NLAB. Our results are comparable to and complement other published series such as the multicentre study by Niazi et al. [28] which gathered data on the use of adjuvant antibiotic loaded bio composite in the management of diabetic foot osteomyelitis and reported infection eradication in 63 patients (90%), with a mean time to ulcer healing of 12 weeks [28]. Vasukutty et al. reported similar results in The Diabetic Foot Journal, with a 94% healing rate and 16 weeks as the average time to wound healing [29].

Cerament use also allows for dead space void filling which is due to the hydroxyapatite particles embedded in the synthetic calcium sulphate carrier [28]. This allows for better wound healing and reduced predisposition to recurrent infections and infection persistence. This was demonstrated in our study with infection persistence demonstrated in only 4 patients who had received adjuvant local antibiotic therapy while in our control group, 15 patients demonstrated infection persistence. This was again observed in our study when both groups were compared, with reinfection rates being higher in the NLAB group and a reduced time to reinfection predisposing patients to increased infection recurrence.

A study published in the Bone and Joint Journal observed the mid- to long-term results of single-stage surgery for patients with chronic osteomyelitis using a bioabsorbable gentamicin-loaded ceramic carrier [30]. The study reported that six patients had recurrent infection while 94% were infection-free [30]. The silo technique for diabetic calcaneal OM treatment was used in 12 patients and outcome was reported by Drampalos et al. in 2018^27^. Complete infection eradication was reported in all 12 of the patients [27].

Our study then examined the amputation rates in both groups which represent failure of overall treatment. The additional use of Cerament was associated with lower treatment failure rates and lower major amputation rates with only 1 patient in group ALB requiring below the knee amputation. Amputations are usually necessitated due to persistence and spread of infection and osteomyelitis.

We then analysed the mortality rates for both groups throughout a 5 year period of time. There was a significant difference in mortality between group NLAB and group ALB, (55.10–12.5%). This could be linked to the prolonged duration of poorer mobility due to infection, persistence of infection and reinfection and the higher amputation rates reported in group NLAB. Amputations in diabetic foot osteomyelitis are linked to increased patient morbidity and mortality. Our data would suggest that adjuvant local antibiotic therapy in diabetic foot osteomyelitis has the potential to alter the natural history and outcomes of this condition. A reduction in the number of surgical procedures was noted in group ALB but on analysis was found not to be statistically significant. Perhaps an earlier introduction of CERAMENT for DFIs treatment could lead to reduced number of procedures and reduced overall hospital stay as suggested by McNally et al. [30]

It is crucial to mention some of the adverse effects most commonly associated with the use of Cerament, such as white discharge from the wound site as the carrier calcium sulphate breaks down [31]. This can often cause some distress and wound maceration but typically resolves in 2–3 weeks [31]. This adverse effect was not encountered in our series. A report by Tarar et al. mentions the risk of iatrogenic hypercalcaemia due to sulphate beads application [32] which was again not encountered by any of the patients in our series. These adverse effects are more likely encountered in association with longer volumes of the product being used but are important to be made aware of.

The limitations of our study include its focus on the management of DFO when the predominant pathogen is gram-positive more notably *Staphylococcus aureus*. There is a potential risk of bias with it being a single-centre study which may not be applicable to other patient populations. It also focuses on local antibiotics as an adjuvant therapeutic agent and not as a standalone treatment modality. While the basic standard of care was applied to both groups, it is pertinent to highlight the existence of several confounding variables that may affect this study. These variables range from the surgeon performing the procedures in relation to our LAB group to the pre-hospital management of our cohort of patients. Our study is also limited by its retrospective nature, but the results are compelling enough to focus more attention on the significant beneficial effects of local antibiotic use in the management of diabetic foot osteomyelitis. Our hospital has now adopted the use of adjuvant local antibiotic therapy as our standard of care for DFO.

## Conclusion

This study offers crucial insights into the promising outcomes linked with the utilization of adjuvant local antibiotic therapy in the form of gentamicin/vancomycin loaded bio-composite bone void filler for managing diabetic foot osteomyelitis. Statistically significant relationships were noted between the use of local antibiotics and enhanced patient outcome, with a three-fold improvement in healing rates, a reduction in disease persistence and reinfection. Adjuvant local antibiotic therapy was associated with a reduction in amputation rates and patient mortality in this group of patients. Our data would support the call for more thorough randomized trials to assess the efficacy and pertinent role that adjuvant local antibiotics may play in association with DFO.

**Table 1:** Table representing patients’ baseline demographics and characteristics within both groups

Variables	Group NLAB (*n* = 49)	Group LAB (*n* = 56)
Age, years	63.21	64.63
Sex:		
Male Female	39 (79.59%) 10 (20.4%)	43 (76.78%) 13 (23.21%)
HbA1c mmol/mol	74.05	76.31
WBC x 10^9^/L	9.34	8.92
Site of infection:		
Forefoot Mid-foot Hind-foot	39 (79.59%) 4 (8.16%) 6 (12.4%)	40 (71.42%) 6 (10.71%) 10 (17.85%)
Surgical procedure:		
Minor amputation (toe amputations) Major amputation (below or above the knee)	21 (42.85%) 6 (12.24%)	15 (26.78%) 1 (1.78%)

**Table 2:** Table representing patients’ outcome within both groups

Outcome	Group NLAB (*n* = 49)	Group LAB (*n* = 56)	*p*-value
Persistent	15 (30.61%)	4 (7.14%)	< 0.00183
Recurrent	24 (48.97%)	11 (19.64%)	< 0.001466
Healed	10 (20.41%)	41 (73.21%)	< 0.0001

## Data Availability

Data is available upon request from the corresponding author.
